# Does Vertical Reading Help People with Macular Degeneration: An Exploratory Study

**DOI:** 10.1371/journal.pone.0170743

**Published:** 2017-01-23

**Authors:** Aurélie Calabrèse, Tingting Liu, Gordon E. Legge

**Affiliations:** 1 Department of Psychology, University of Minnesota, Minneapolis, Minnesota, United States of America; 2 Department of Ophthalmology and Vision Science, Eye and ENT Hospital of Fudan University, Shanghai, China; Kobenhavns Universitet, DENMARK

## Abstract

Individuals with macular degeneration often develop a Preferred Retinal Locus (PRL) used in place of the impaired fovea. It is known that many people adopt a PRL left of the scotoma, which is likely to affect reading by occluding text to the right of fixation. For such individuals, we examined the possibility that reading vertical text, in which words are rotated 90° with respect to the normal horizontal orientation, would be beneficial for reading. Vertically oriented words would be tangential to the scotoma instead of being partially occluded by it. Here we report the results of an exploratory study that aimed at investigating this hypothesis. We trained individuals with macular degeneration who had PRLs left of their scotoma to read text rotated 90° clockwise and presented using rapid serial visual presentation (RSVP). Although training resulted in improved reading of vertical text, the training did not result in reading speeds that appreciably exceeded reading speeds following training with horizontal text. These results do not support the hypothesis that people with left PRLs read faster with vertical text.

## Introduction

People suffering from macular degeneration (MD) often lose the ability to use central vision. Both age-related macular degeneration (AMD) and juvenile forms of macular degeneration (JMD) can lead to the development of bilateral central scotomas, seriously affecting the performance of high-resolution tasks such as reading. Difficulty with reading is often the primary complaint of people with central vision loss [1].

Despite recent advances in the treatment of wet AMD [2], MD continues to be a leading cause of severe visual impairment in developed countries--AMD is expected to affect 288 million people worldwide by 2040 [3]. Since no effective treatment is available to restore normal central vision, it is essential to optimize the capabilities of peripheral vision for reading. For this reason, there is research interest in designing training methods to improve peripheral reading abilities.

In the presence of a bilateral central scotoma, many individuals with MD develop an eccentric preferred retinal area to substitute for the function of the fovea [4, 5]. This area is called the Preferred Retinal Locus (PRL). The PRL may be located vertically (above or below) relative to the scotoma or lateral (left or right) to the scotoma. A majority of individuals with MD adopt a lateral PRL, and more specifically, a left-field PRL [6]. For English speakers who are used to reading horizontal text, lateral placement of the PRL may be disadvantageous for several reasons.

**Fig 1:**
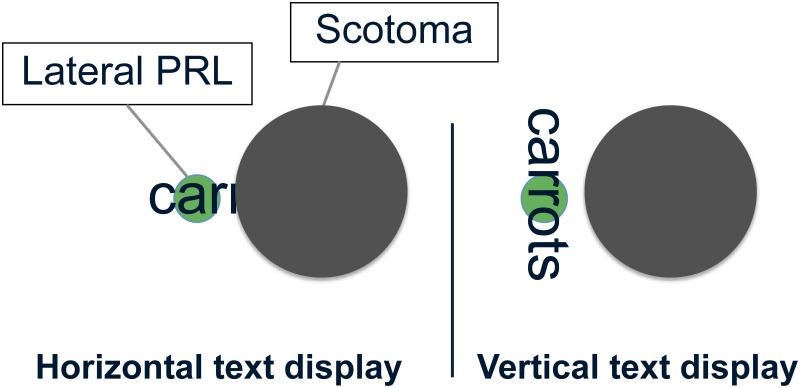
Illustration of the spatial relationships between text, central scotoma and PRL for an individual using a left PRL. The green circle indicates visual-field location of fixation (PRL), oriented to the left of the scotoma. With regular text display—horizontal (left panel)–the word is partially occluded by the scotoma. Rotating the text by 90° –vertical (right panel)–allows the word to be entirely visible within one fixation.

First, when reading horizontal text using a lateral PRL, the central scotoma may mask part of the text, allowing fewer letters to be visible at one glance (Fig 1). Cheong et al. (2008) [7] measured visual spans (the number of adjacent letters that can be recognized reliably without moving the eyes) for participants with macular degeneration. They showed that for lateral PRLs, the size of the visual span is indeed decreased by the presence of the scotoma. It is hypothesized that reading speed is determined in part by the size of the visual span [8, 9]: a reduced visual span increases the reading time (and therefore, decreases reading speed) by increasing the number of fixations [10]. We might expect people with PRLs lateral to a scotoma to have a smaller horizontal visual span, and as a result, to experience slower reading speed with horizontal text. Additionally, left PRLs are expected to be even more detrimental than right PRLs for English readers, for whom text is read from left to right, because the scotoma occludes the upcoming words. This left PRL disadvantage has been shown before in studies with simulated scotomas [11, 12].

Second, Peli (1986) [13] proposed that reading eye movements are more effective orthogonal to the line between the fovea and PRL i.e., in the vertical direction for lateral PRLs. In this case, horizontal text would not lead to the most efficient eye movement patterns.

Based on these observations, we hypothesize that individuals with macular degeneration using a PRL to the left of the scotoma, might ultimately read vertical text better than horizontal text. Vertically oriented text would be tangential to the scotoma (Fig 1), avoiding occlusion of upcoming text by the scotoma, and would afford the possibility of a larger visual span.

Despite this theoretical advantage for reading vertical text for participants with MD, it is likely that a lifetime of experience with horizontal text may be a disadvantage for vertical text reading performance. Yu et al. (2010) [14] tested normally sighted participants reading with conventional horizontal text and three arrangements of vertical text—upright letters arranged vertically (marquee), and horizontal text rotated 90° clockwise or counterclockwise. On average, reading speed for horizontal text was 139% faster than marquee and 81% faster than the rotated texts. Visual spans were smaller for vertical arrangements of letters. Similarly, horizontal reading speed is faster than vertical reading speed by about the same ratio in normal peripheral vision although both horizontal and vertical reading are slower than in central vision [15]. It remains to be determined if practice with vertical reading would offset this lifetime of experience with horizontal text, and potentially provide benefits to MD subjects.

Gibson (1963) [16] defined perceptual learning as ‘‘[any] relatively permanent and consistent change in the perception of a stimulus array, following practice or experience with this array.” It has been shown that reading speed in normal peripheral vision can improve with training through perceptual learning [17, 18]. Perceptual learning, based on training with RSVP text, has also been shown to yield benefits in reading speed in macular degeneration [19]. Subramanian et al. (2014) [15] have shown that perceptual learning can be used to close the gap between vertical and horizontal reading speed in normal peripheral vision. They showed that, four days of practice at one hour per day in reading vertically oriented text resulted in reading speeds similar to horizontal reading speeds. These results with normal participants encouraged us to expect that MD participants could overcome initial deficits in vertical reading through practice.

Further encouragement comes from studies with Japanese readers who are accustomed to reading both horizontal and vertical text. Matsumoto et al. (2005) [20] showed that some participants with bilateral macular disease shifted from a PRL above or below the scotoma for horizontal reading to a lateral PRL for vertical reading. Further evidence of the superiority of vertical text was also shown in Japanese readers with central field loss [21]: depending on the size of the scotoma and the position of the PRL, some individuals read vertical text faster than horizontal text. These findings increase the likelihood that vertical text could be advantageous for some people with macular scotomas.

Following up on Subramanian et al. (2014) [15], who demonstrated the potential of training normally sighted participants to read vertically oriented text, we investigated vertical reading in individuals with central field loss. In this paper, we present the results of an exploratory study to determine: 1- if MD participants with left PRLs can learn to read vertical text, and if so 2- whether such improvement yields faster reading speed than conventional horizontal text.

To investigate these questions, we trained two groups of participants with macular degeneration, all of whom had well-established left PRLs. Left PRLs are the primary target because they are very common and would likely be more detrimental to reading than right PRLs or PRLs above or below the scotoma. In this sense, we could expect MD subjects with PRLs left of the scotoma to benefit most from vertical reading. One group of participants was trained to read RSVP sentences displayed horizontally, while the second group was trained to read RSVP sentences displayed vertically (words rotated 90° clockwise). By optimizing text presentation [22] (ie. adjusting reading orientation to PRL placement), we sought to determine whether a functionally relevant reading-speed improvement would be realized.

## Methods

### Participants

Ten participants with a diagnosis of macular degeneration were recruited for this exploratory study—8 with AMD and 2 with JMD from Stargardt’s disease. Each participant had a bilateral central field loss and no history of neurologic disease. Participants were all native English speakers with no known reading disabilities prior to their eye condition. All had functionally useful ability to read magnified print. All testing was administered with the participants wearing their typical near-viewing lens correction prescribed within 6 months of enrollment. Participants gave written informed consent in accordance with procedures and protocols approved by the University of Minnesota Institutional Review Board and following the tenets of the Declaration of Helsinki.

### Clinical assessment

Prior to the training, each participant visited our lab at the University of Minnesota to complete a series of primary tests including measures of distance visual acuity (ETDRS letter chart [23]) and contrast sensitivity (Pelli-Robson Contrast Sensitivity chart [24].). Reading performance (Maximum Reading Speed and Critical Print Size) was assessed using the MNREAD acuity chart [25, 26]. Unimpaired cognitive status was confirmed with the Mini-Mental State Exam (MMSE), all subjects exceeding a criterion score of 27 on this test. Participants also completed oral questionnaires regarding their eye condition, and reading habits.

Characteristics of the central scotomas and participants' fixation were assessed using the microperimeter MP-1 (Nidek). Both eyes of each participant were tested monocularly, the other eye being patched. Static perimetry was first performed in order to assess the position and the size (in square degrees) of each scotoma: participants were asked to fixate at all times on a red cross projected in the middle of the viewing area. The fixation cross size was set to either 2° or 4°, depending on the participant’s abilities. In the mean time, a series of Goldmann V stimuli were displayed (200 ms each) on the screen. Unseen stimuli correspond to the position of the functional scotoma. A fixation exam was then performed to estimate the position of the preferred retinal locus (PRL) used to fixate and the stability of fixation. Each participant was asked to fixate on the target for 25 seconds (as recommended by the manufacturer). Fixation stability was defined as the bivariate contour ellipse area (BCEA) encompassing 68% of fixation positions [27, 28]. The centroid of the fitted ellipse was used to estimate the location of the fixation PRL [29].

To be included in the study, participants had to have: 1- an absolute central scotoma in each eye and; 2- an established lateral PRL in each eye. Table 1 summarizes the results of visual tests, MNREAD and microperimetry examinations for each participant. All 10 participants used a left PRL when fixating on a static target with their better eye and either a left or a right PRL when fixating on a static target with their worse eye.

**Table 1:** Demographic and clinical characteristics of our ten participants.

Participant ID		S1	S2	S3	S4	S5	S6	S7	S8	S9	S10
Gender		F	F	F	M	M	F	F	F	F	M
Age		88	72	86	56	49	89	82	92	87	94
Diagnosis		AMD	AMD	AMD	Stargardt	Stargardt	AMD	AMD	AMD	AMD	AMD
Visual acuity (logMAR)	OD	**1.64**	1.22	**1.14**	0.94	1.54	1.54	1.00	**0.76**	0.90	**0.52**
	OS	LP	**1.14**	1.58	**0.94**	**0.92**	**1.22**	**0.82**	1.04	**0.22**	1.02
	OU	1.56	1.14	1.16	0.80	0.94	1.16	0.94	0.86	0.24	0.46
Log Contrast sensitivity		0.15	0.75	1.20	1.35	1.30	0.10	0.70	1.15	0.85	1.35
Maximum Reading Speed (wpm)		60	14	30	90	50	15	35	65	70	85
Critical Print Size (logMAR)		1.30	1.70	1.30	1.20	1.22	1.50	0.80	1.50	0.60	1.32
Scotoma size (deg^2^)		64.8	49.3	301.4	78.5	122.5	380.0	326.8	221.6	226.9	37.7
PRL position (relative to the scotoma)		left	left	left	left	left	left	left	left	left	left
PRL eccentricity (deg)		6.05	6.99	9.96	7.95	10.04	8.44	5.53	5.05	---	6.24
Fixation stability—BCEA (minarc^2^)		47210	17110	16935	15963	36096	13672	4067	---	---	13418

Visual acuity was measured monocularly in both eyes and binocularly. Contrast sensitivity and MNREAD measurements were obtained under binocular conditions. Microperimetry results are reported for the better eye only [30], defined by the better visual acuity. When acuity was the same in both eyes, the better eye was defined as the one having the smallest PRL eccentricity (distance between the fixation PRL and the fovea [31]). Visual acuity of the better eye is reported in bold. LP stands for ‘light perception’.

### Training procedure

Each participant was trained using a home-based training protocol, designed in our laboratory. Conducting the training at home reduced the participant’s burden in time and difficulty in coming to our lab on campus for multiple sessions. A member of our lab staff delivered and set up a laptop computer for testing at the participant’s home. All the pre- and post-training measurements, as well as the training sessions, were conducted remotely, with the participant at home and the experimenter in the lab. Remote control software and 3G wireless network connection were used to run the tests, allowing the experimenter to launch programs and trigger each trial remotely. Participants did not have to operate the laptop, except from a single press on the power button before each session. Live communication was established through Skype. At the beginning of each session, the experimenter and participant went through a checklist to confirm arrangements for lighting and viewing distance.

**Fig 2:**
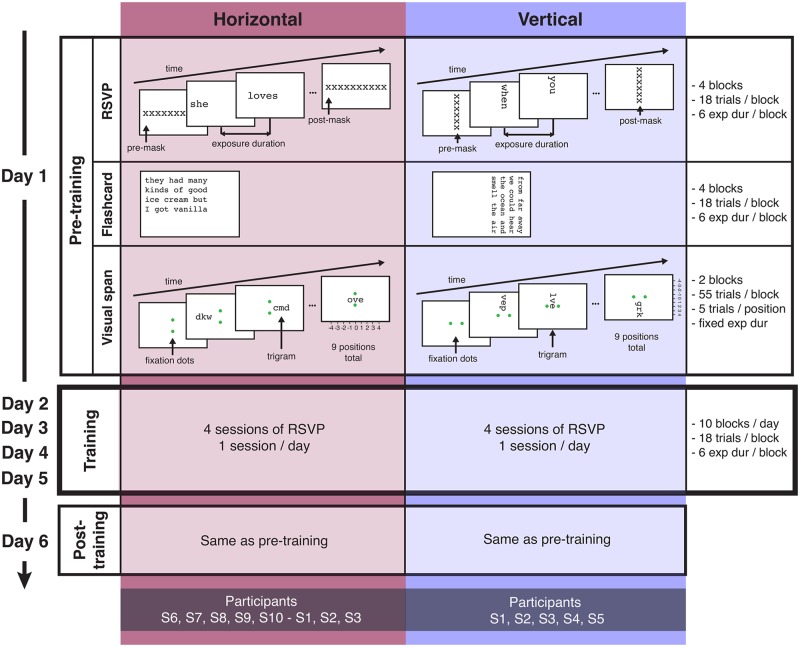
Testing procedure.

Six sessions (1 session per day; spread over the course of two weeks) were necessary to complete the experiment. Each session lasted 60 to 90 minutes (including rest breaks). The actual training took place from Day 2 to Day 5. Prior to training, the baseline performance of each participant was assessed during a ‘pre-training’ session (Day 1). After training, a ‘post-training’ session (Day 6) was run to estimate training-related improvement (Fig 2). ‘Pre-training’ and ‘post-training’ measurements included: 1) RSVP reading speed, 2) flashcard reading speed (to check for transfer to page reading, involving eye movements), and 3) visual span size (to see if training would also produce an increase in the size of the visual span).

Participants were either assigned to vertical or horizontal training. Participants S1, S2, S3, S4 and S5 were trained with vertical text display (text rotated 90° clockwise). Participants S6, S7, S8, S9 and S10 were trained with horizontal text display. Three to five months later, three participants who had received vertical training, underwent horizontal training to get a direct comparison between training with vertical and horizontal text displays. Because of practical issues, only S1, S2 and S3 completed this cross training.

All training was conducted with RSVP reading. This task has been shown to yield better reading speed improvement than other types of training with letters and words [14].

### Apparatus, stimuli and outcome measures

Visual stimuli were presented binocularly at 25 cm on a 17” Dell laptop (Dell Precision M6500). Stimuli were generated and presented using MATLAB 7.10 software (MathWorks, Inc.) with Psychophysics Toolbox extensions [32, 33, 34]. All sentences and letters were displayed in Courier font with high-contrast black letters on a white screen (luminance of 190 cd/m^2^). Print size was adjusted for each participant to twice their MNREAD critical print size. Sentences (mean = 11.5 words each, SD = 0.67) presented during the RSVP and flashcard tasks were drawn from two different pools of sentences [35, 36], with no sentence presented more than once to a given participant.

#### RSVP reading

Sentences were presented on the screen one word at a time, either oriented horizontally or vertically (rotated 90° clockwise). For a given orientation, words were all displayed at the same location (aligned on the first character), requiring minimal eye movements (Fig 2). Participants were asked to fixate at all times using their PRL and read the sentences aloud. Sentences were presented during blocks of 18 trials including 6 different exposure durations. The range of word exposure durations was chosen to yield an overall percent correct located between 10 and 100%. This range was adjusted over the course of the experiment to match the participant’s change in performance. In each block of trials, the proportion of words read correctly was calculated for each exposure duration. A cumulative Gaussian function was then fitted to the data. The exposure time yielding 80% correct word recognition was estimated from the fitted function and converted to reading speed in words per minute (wpm). During the training, we aimed to present 10 blocks of 18 sentences each during each session, but this number varied slightly depending on the participant’s reading speed.

#### Flashcard reading

Sentences were displayed as a whole on the screen, either oriented horizontally (first word starting at the left top corner) or vertically (first word starting at the right top corner). Sentences were displayed over four lines to simulate page reading, which requires saccadic eye movements (Fig 2). Data collection and analysis were similar to RSVP testing. Blocks of 18 sentences were presented to the participant at 6 different exposure durations. The range of sentence durations was chosen to encompass reading performance from 10 to 100% correct and was modified if needed during the experiment, based on the participant’s change in performance. For each block, a cumulative Gaussian function was fitted according to the proportion of words correctly read. From this function, we estimated the exposure time yielding 80% correct word recognition and converted this value to reading speed in words per minute (wpm).

#### Visual span

Participants were asked to fixate between two dots at the center of the screen while random strings of three letters (called trigrams) were presented at various eccentricities (Fig 2). Participants were asked to report all three letters from first to third while maintaining fixation. Visual span size was then calculated with a standard method, based on the recognition accuracy for nine letter positions centered at fixation [37]. For horizontal measurements, we used a standard display [7]: two dots, vertically arranged around the horizontal midline, were used as a fixation target. Trigrams of horizontal letters were randomly presented at various distances left and right of fixation along the midline. For vertical visual span, the same display was generated, but it was presented 90° clock-wise rotated. For the visual span measurement to be effective, the participant needs to maintain good fixation. Fixation was not recorded at home, and we used the MP1 measurement to estimate the ability of the participants to keep fixating between the dots. Based on the value of their BCEA (Table 1) and the shape of the ellipse, we determined that each participant’s fixation accuracy was within two letters for the print size used in testing.

## Results

### MD participants with left PRLs can learn to read vertical text

Participants S1 to S5 were trained with RSVP sentence reading, with the words rotated 90° (Fig 2). There were three outcome variables, measured in the pre- and post-tests—RSVP reading speed for vertical text, flashcard reading speed for vertical text, and visual-span measured in the vertical direction. All three of these measures had larger values in the post-test.

**Fig 3:**
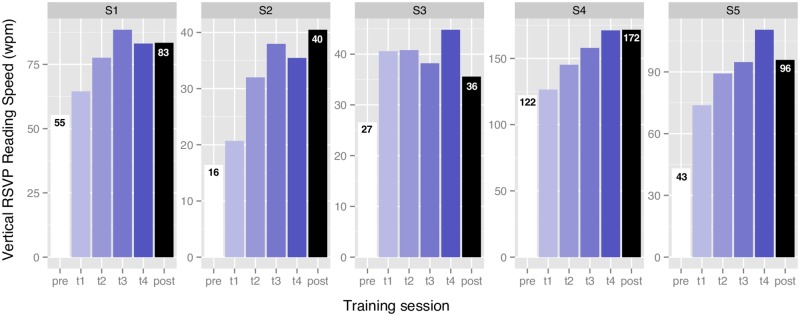
Changes in RSVP reading speed through the course of vertical training. Data are shown for each of the four training sessions (t1 to t4), plus the pre- and post-training sessions. Except for S3 who experienced a plateau in performance after the first training session, all participants continued to improve throughout the training.

RSVP reading speed for vertical text (Fig 3) increased by a mean of 79% (median 51%, range 33% to 150%). The largest percentage improvement in reading speed was for S2 whose vertical reading speed increased from 16 to 40 wpm (150% improvement). The smallest improvement was for S3 whose vertical reading speed increased from 27 to 36 wpm (33% improvement) (Fig 3). Expressing such improvements in percentage gives a sense of the functional benefit experienced by the participants.

**Fig 4:**
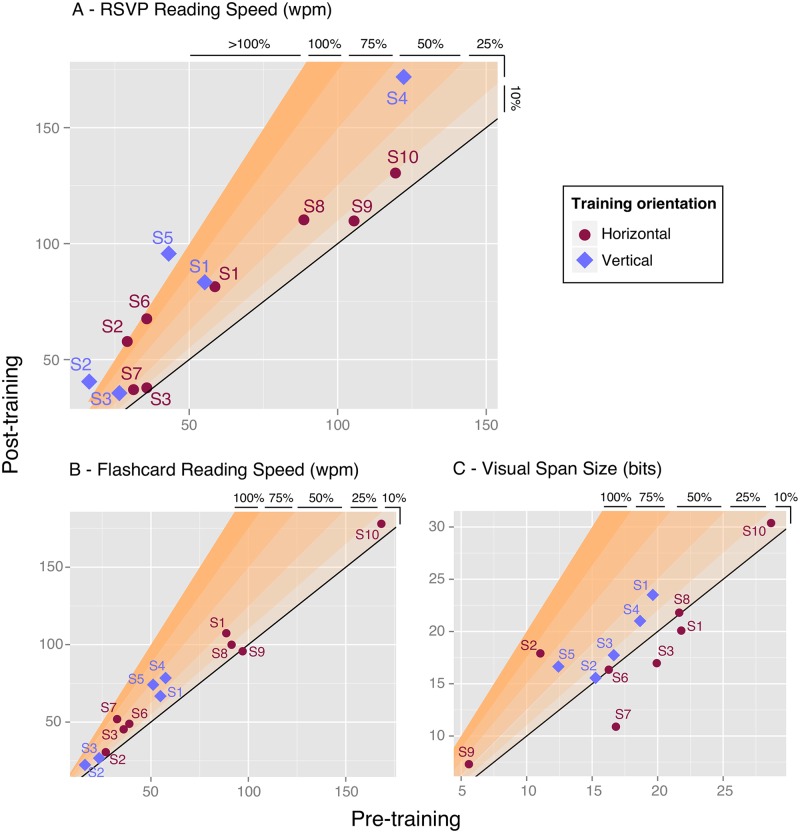
Outcome measures before and after training: A—RSVP reading speed; B- Flashcard reading speed; C—Visual Span size. X-axis shows the pre-test performance, Y-axis shows the post-test performance. Data above the black line indicates better performance following training. The range of improvement (in %) is color-coded in orange.

Both flashcard reading speed and visual span size also increased after vertical training (Fig 4). Vertical flashcard reading speed increased by a mean of 31% (median 37%, range 14% to 45%), showing some partial transfer of improvement to an everyday reading task. All 5 participants had larger vertical visual spans after training, with a mean increase of 2.37 bits ie. 15% (median 13%, range 2% to 34%). This increase in visual span is consistent with the hypothesis that reading speed is associated with the size of the visual span [10].

### How does vertical training improvement compare with horizontal training?

Participants S6 to S10 were trained with RSVP text in the horizontal orientation. Overall, the horizontal RSVP reading speed increased by a mean of 29% after training (median 18%, range 4% to 89%). This improvement is quantitatively smaller than the 79% reported above for the vertical training. Fig 4 shows the relationship between pre- and post- performance for all participants on the two orientations.

All five participants, trained with horizontal text, showed very little change, no change or even a decrease in visual span size after training (Fig 4C). By comparison, all participants trained with the vertical text orientation showed an increase in the size of their visual spans (as reported in the previous section). This difference in the effects of training on the visual span may not be surprising given that all participants used a lateral PRL to fixate during the visual span measurement. For the horizontal training, the size of the visual span is limited by the adjacent scotoma.

### Does vertical training yield faster reading speed than conventional horizontal training?

Participants S1, S2 and S3 were trained with both vertical and horizontal text. Comparing their performance before and after both types of training gives a direct assessment of the potential advantage for vertical training when using a lateral PRL. First, like normally sighted participants, these three participants began reading RSVP vertical text more slowly than horizontal text (Fig 4A). On average, vertical pre-training reading speed was 39% lower than horizontal pre-training reading speed. This finding is consistent with recent results from Subramanian et al. (2014) [15] who found a significant difference of 37% between horizontal and vertical eccentric reading speeds in a group of normally sighted subjects. Second, after training, the vertical RSVP reading speed of all three participants matched or exceeded their untrained horizontal reading speeds. The greatest difference was for S1 and S2 whose vertical post-training reading speeds were respectively 40.6% and 38% faster than their horizontal pre-training reading speeds (83 to 59 wpm, and 40 to 29 wpm respectively). Participant S3, on the other hand, showed no difference at all between horizontal pre-training and vertical post-training reading speeds (36 wpm in both conditions). Third, following both horizontal and vertical training, all three participants showed slower or similar RSVP reading speed for vertical text compared with horizontal text. S1 showed a slightly faster vertical reading speed (2% difference); S3 showed a somewhat slower vertical reading speed (6% difference); and S2 showed considerably slower vertical reading speed (43% difference).

## Discussion

This exploratory study focused on two questions. First, would reading performance of MD subjects with left PRLs exhibit increased reading speeds when trained to read vertically oriented text? The answer is yes. We found that RSVP training with vertical text improved vertical reading performance by an average of 79% (SD = 52%). This value is higher than the average improvement of 29% (SD = 35%) measured in our participants with left PRLs who were trained to read horizontal text. By comparison, Chung (2011) [19] found a 53% average improvement in reading speed by a group of six MD subjects trained with horizontal RSVP text. The fact that participants continue to improve over the course of the vertical training, implies that the performance change measured after vertical training cannot simply be du to task familiarity. We conclude that individuals with MD can learn to read vertically oriented text.

Second, we asked how the benefits of vertical training compare to the benefits of horizontal training for MD subjects with lateral PRLs. We trained three participants with both orientations and found that their post-training performance with vertical text was either slower or similar to their performance after training with horizontal text. As a second way of comparing the likely benefits of vertical and horizontal training, first note that our results, and those of Subramanian et al. (2014) [15], show that the pre-training baseline reading speed for vertical text is approximately 60% of the pre-training horizontal reading speed. Second, our data indicate that training yields about an 80% increase in vertical reading speed. Combining these two effects, we would expect the post-training vertical speed to exceed the pre-training horizontal speed by only about 8%. This value is substantially smaller than the 29% improvement we found for our group trained with horizontal text and the mean improvement of 53% found by Chung (2011) [19]. In short, taking the difference in baseline reading speeds into account, the greater percentage improvement in vertical reading speed following training is not sufficient to exceed the expected benefits from horizontal training. Performance is known to vary consistently at isoeccentric locations in the visual field. The general advantage of visual processing at the horizontal meridian is usually called Horizontal–Vertical Anisotropy [38]. Specifically, at a fixed eccentricity, performance is better along the horizontal meridian than the vertical meridian. This asymmetry might help explain why, despite the occluding presence of the scotoma, horizontal text is easier to process than vertical text.

Subjectively, all participants trained with the vertical text said that they still preferred standard horizontal reading. Only one of them (S5)–the youngest of the group, 49 years old—commented after training that he would now use vertical orientation as a side strategy when horizontal text became hard to read (for example when reading his mail, he would turn the page 90° and read words oriented vertically) but would still rely primarily on reading horizontal text.

In conclusion, this exploratory study does not support the idea that training to read vertical text would provide major benefits for people with MD who are accustomed to reading horizontal text and who have left PRLs. However, this conclusion is based on a small number of subjects and would benefit from a larger and more diverse sample. For example, it would be interesting to use vertical training on young individuals with MD, with potentially greater plasticity than the present participants (mean age = 79 years old). Finally, although we would expect MD subjects with left PRLs to benefit most from vertical reading, it would be informative to also test subjects with right PRLs.

There are two caveats concerning our PRL measurements. First, we measured PRL locations with a fixation task. Previous studies have shown that the location of the fixation PRL is not a significant predictor of the reading rate of patients with central field loss [31, 39, 40, 41], possibly indicating that the PRL used during fixation is different from the PRL used in reading. Second, our PRL measurements were conducted monocularly while the reading tasks were binocular. Future investigation would benefit from a recently designed method allowing measurement of binocular PRLs with the MP1 [42].

Lastly, the question whether the current approach may benefit more individuals who are accustomed to reading vertical text, like Japanese readers, remains open.
